# Nano-Bio-Technology and Sensing Chips: New Systems for Detection in Personalized Therapies and Cell Biology

**DOI:** 10.3390/s100100526

**Published:** 2010-01-12

**Authors:** Sandro Carrara

**Affiliations:** Swiss Federal Institute of Technology-Lausanne, (EPFL)-EPFL IC ISIM LSI1-INF 338 (Bâtiment INF), Station 14 CH-1015 Lausanne, Switzerland; E-Mail: sandro.carrara@epfl.ch; Tel.: +41-21-693-0915; Fax: +41-21-693-0909

**Keywords:** biochip, DNA, enzymes, cytochromes, antibodies, carbon nanotubes, gold nano-particles, ethylene-glycol monolayers

## Abstract

Further advances in molecular medicine and cell biology also require new electrochemical systems to detect disease biomarkers and therapeutic compounds. Microelectronic technology offers powerful circuits and systems to develop innovative and miniaturized biochips for sensing at the molecular level. However, microelectronic biochips proposed in the literature often do not show the right specificity, sensitivity, and reliability required by biomedical applications. Nanotechnology offers new materials and solutions to improve the surface properties of sensing probes. The aim of the present paper is to review the most recent progress in Nano-Bio-Technology in the area of the development of new electrochemical systems for molecular detection in personalized therapy and cell culture monitoring.

## 1. Introduction

One of the main objectives in personalized therapy is to inject drug doses in the right amount with respect to a patient’s metabolic conditions. This is a key-point because patients can express depleting isoforms of cytochrome P450 accordingly to their genotype. Cytochrome P450 is a central protein in human metabolism. It has been already proven that different patient's genotype groups present different amounts of mean plasma concentration after injection of the same amount of drug [1]. For that purpose, Roche has developed a genetic test called AmpliChip [2]. The Roche test may detect depletion of the two genes related to protein expression of the 2D6 and 2C19, which are different isoforms of the cytochrome P450. The AmpliChip received FDA approval and it is now on the market. Although it is a powerful tool to identify four different patient’s classes, the test identifies their genetic predisposition to metabolize drugs catalyzed by only these two P450 isoforms, while the human metabolism involves more than three-thousand different P450 isoforms. Moreover, human metabolism is not only related to genetic predisposition, but also to (varying) daily conditions of the patients. A proof of that complex situation is that only 20–50% of patients receive any benefit from therapies where most effective compounds are employed [3]. Nowadays, therapeutic drug monitoring is possible only in specialized laboratories, and it requires large equipments and clinical feedback is only available after several days, so new point-of-care or portable technologies are absolutely required for monitoring drug metabolism in blood or in serum in order to proceed forward in personalization of the therapies.

Cell therapy and regenerative medicine are other highly innovative branches of modern medicine. In some cases, damaged tissues may be replaced by using engineered ones obtained from stem cells [4]. To that end, new automated factories are under development in order to improve the fabrication processes of such engineered tissues [5]. New molecular compounds have been investigated to improve cell feeding [6]. New tools for cells sorting are under development using magnetic fields as driving forces [7]. Electric fields were investigated as further physical parameters pushing differentiation toward electrically specialized cells [8]. However, many biochemical mechanisms taking place during cell differentiation are still missing. Therefore, a deep understanding of cell metabolism during differentiation is highly required to clarify many details in stem cell biology and to provide improved control in tissue engineering.

Microchip technology may provide new circuits and systems to address these arising demands. Implantable biosensors for glucose monitoring [9], label-free biochips for DNA detection [10], point-of-care devices for drug testing in saliva [11], and for glucose measurements in cell cultures [12] are good examples. However, more often sensitivity is not in the right range, specificity is poor, and the proposed systems are not stable enough for real-time applications. Therefore, new efforts are required to improve biochip performance. Nanotechnology may provide new materials and solutions to enhance biochip characteristics.

“Biology is not simply writing information; it is doing something about it. A biological system can be exceedingly small. Many of the cells are very tiny, but they are very active” said Richard Phillip Feynman in his famous lecture on Nanotechnology at Massachusetts Institute of Technology in 1959. According to him, nanotechnology should learn from biology. So, the best “Nanotechnology” seems to be the “Nano-Bio-technology”, which also provides new opportunities to improve nano-bio-chips, *i.e.*, new bio-materials fabricated with control at the nano-scale.

In this paper, biological and organic structures at the nanometer scale will be considered as building blocks. Their working advantages in the field of biochips will be demonstrated by considering some examples. 2D, 1D, and 0Dimensional systems made with these building blocks will be conceptually discussed and their experimental investigations will be briefly summarized. Advantages provided by these nano-structures will be evaluated by means of comparisons with bulk materials. Applications to detection in metabolism, cancer markers, and DNA will be used to show enhanced performances due to nano-bio-technology. The biophysics of the related interfaces between sensing surfaces and biological samples will be deeply argued. Successful examples will be used to show increased sensitivity, specificity, and detection capability. Some new and innovative ideas will be briefly presented about applications of nano-bio-chip in personalized therapies and cell biology.

## 2. 2D Nano-Structures Improving DNA-and Immuno-Chip

**Figure 1.**
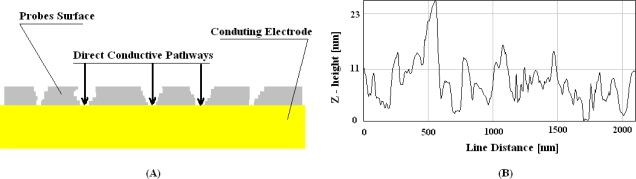
(A) A model describing nano-scale groves that provide direct conducting pathways through the whole probe film. (B) AFM microscopy profile of groves registered on a surface of ss-DNA probes directly immobilized onto gold.

Referring to the fabrication of nano-structures, a 2D system may be obtained by keeping one of its dimensions in the nano-scale. For example, a surface can be conceptually thought of as a cube with a height equal to zero in the z-dimension. Thus, a 2D nano-structure might be seen as a sheet of material where one dimension is in the nano-scale while the other two are in the micro or millimeter scale. Good examples are molecular mono-and multi-layers built by using Langmuir-Blodgett [13] or Self-Assembly [14] techniques. In the first case, an ordered mono-molecular layer is firstly obtained at the air/water interface and then transferred onto a solid substrate. By repeating this step, multi-layers may be assembled. A highly ordered structure at the nano-scale may be obtained by intercalating proteins and amphiphilic molecules (such as fatty acids or alkanethiols) [15]. In the case of self-assembly, a molecular layer may be obtained by leaving substrates into molecular solutions over-night, and enabling the molecules to form stable chemical bonds onto the substrate’ surfaces. An ordered nano-scale structure may be obtained by intercalating both proteins and amphiphilic molecules [16], too. In both the cases, multi-layers may include both protein functionalities and amphiphilic features and they may provide improved specificity and more reliable performance in biochips for DNA or antigen detection. More often, published papers related to label-free detection present systems that lack in specificity and reliability. A label-free technology largely proposed for fully-electronics DNA detection consists in measuring capacitance or impedance variations upon DNA hybridization onto a sensing surface. This technique has been initially proposed for antigen [17], and then for DNA [18] detection. VLSI architectures were proposed for fully-electronic readers in DNA-Chips [10]. However, specificity was not very high [10,19], reproducibility between electrodes was close to 40% [19], detection errors were comparable with signal amplitude [10], data points were largely scattered [17,18], and time-series presented very large time-drifts [17,20]. All those serious drawbacks were related to not well insulated probe surfaces. It has been shown that nano-sized grooves crossing the film are related to capacitance time-drift [21] and they provide conducting pathways, which affect the ideality of the electrochemical interface [22]. Figure 1 (A) schematically shows the profile of such grooves on a probe surface, while Figure 1 (B) displays the film profile at the nano-scale of a probe surface obtained by thiolated ss-DNA directly immobilized onto gold by following well known procedures [23]. The AFM profile clearly shows deep grooves crossing the whole film thickness. Such grooves represent nano-scale apertures through the probe film, which allow solution ions to penetrate into the film and directly discharge at the electrode surface.

**Figure 2.**
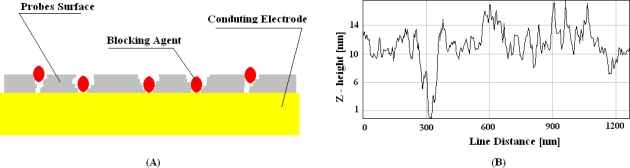
(A) A model describing diminished nano-scale groves by using blocking agents to close direct conducting pathways in probe film. (B) AFM microscopy profile of lesser frequent groves registered on a surface of ss-DNA probes onto gold co-immobilized with lipoate-molecules.

This phenomenon results in two main drawbacks for detection: first, it shows an undesired behavior in terms of film resistivity, which also affects the frequency-dependence behavior of the film capacitance [22]; second, it provides an unstable electrochemical interface that affects time-stability of capacitance measurements [21]. To avoid these two effects, different strategies have been proposed. The most established technique is to close conducting pathways by using some blocking agents, as shown schematically in Figure 2(A).

It can be done in two different ways: by post-treatment of probe surfaces with blocking agents or by a co-immobilization of both probing and blocking molecules. The most popular blocking agent suggested in literature is mercaptohexanol, which has been widely used in the past. However, tests with redox reactions of potassium ferrocyanide on such surfaces clearly show that post treatment does not result in insulated surfaces, even if the blocking agents are the longer 1-dodecanethiols (see Figure 2 in reference [18]). Thus, new kinds of blocking agents have been recently proposed in order to improve reliability of probe films in capacitance DNA detection. It has been demonstrated that the number of grooves is largely reduced if probes are co-immobilized with lipoic acids [23].

**Figure 3.**
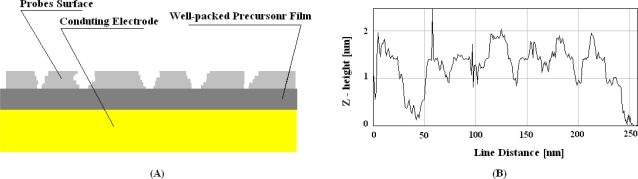
(A) A model describing the absence of nano-scale grooves in a well-packed mono-layer precursor formed below the probes film. (B) AFM microscopy profile registered on a well-packed precursor mono-layer obtained with ethylene-glycol alkanethiols immobilized onto gold.

**Figure 4.**
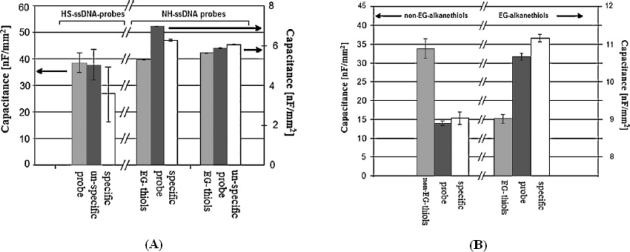
(A) Direct comparison between detection performances of ss-DNA probes directly immobilized onto gold and ss-DNA probes immobilized onto ethylene-glycol mono-layer. (B) Direct comparison between detection performances of antibody probes immobilized onto mono-layers without or with ethylene-glycol function (both figures are reprinted from reference [26] with permission from Elsevier, copyright 2008).

Such molecules present two sulfur groups suitable for a strong anchorage of blocking agents onto gold surface. Moreover, the use of diethanolamines for the other side of this new blocking agent provides more hydroxyl groups, which are able to improve the stability of the electrochemical interface by coordinating and stabilizing more water molecules of the sample. The co-immobilization of ssDNA probes with lipoate-diethanolamines results in a highly diminished number of deep grooves, as shown in Figure 2(B), and it results in lower acquisition errors during measurement [23]. However, grooves crossing the film are detectable even in that case, as clearly demonstrated by the large AFM tip deflection reported in Figure 2(B) up to the value of 300 nm in the line distance. So, much more densely packed films are required to avoid such grooves in the probe films, as schematically shown in Figure 3(A). It has been already demonstrated that alkanethiol films do not allow stable capacitance measurements in time, even though they are made by molecules with alkyl chains as long as eleven methylene groups [17]. These films present deep grooves crossing the whole molecular structure [21], too. Stable capacitance measurements have been indeed registered by immobilizing probes onto precursor films formed with sixteen methylene groups [17,24], or formed with alkanethiols with eleven methylene groups and three ethylene-glycols [25]. It has been proven that mono-layers obtained by using such ethylene-glycol alkanethiols do not present any grooves and the average corrugation registered with AFM is very close to that registered on the substrates [21], as shown by figure 3(B). Such innovative ethylene-glycol monolayers were originally proposed to improve specificity in label-free protein-based SPR detection [16]. More recently, they have been proposed to obtain highly insulated surfaces (see Figure 9 in reference [25]). They also provide highly stable-in-time electrochemical properties [21]. These precursor films were very recently proposed to obtain innovative ss-DNA probe surfaces too [26]. In particular, it has been proven that ethylene-glycol functions improve the time stability of the electrochemical probe surface, and enhance detection specificity. The improved performance of such innovative ethylene-glycol films are reported in Figure 4. Different immobilization strategies are compared in terms of probe performance. Detection errors are almost negligible when DNA probes are immobilized onto the ethylene-glycol film, as shown in Figure 4(A). The detection errors are much smaller when antibody probes are immobilized onto the ethylene-glycol film than when they are immobilized onto non ethylene-glycol precursors. The capacitance value is reduced by one order of magnitudes in the case of ethylene-glycol because of a longer molecular chain. Worth noting is the fact that capacitance changes result in the opposite direction after probe immobilization onto films with or without ethylene-glycol functions. This is due to the different amphiphilic characters of glycol groups compared with methylene groups.

## 3. 1D Nano-Structures Improving Enzyme-Chips

**Figure 5.**
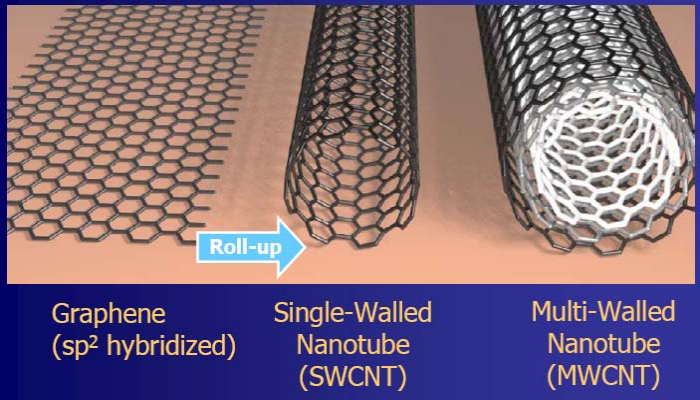
Schematic model describing single-walled and multi-walled carbon nanotubes conceptually obtained from single graphene sheets (courtesy of K. Banerjee/California University, Santa Barbara).

A 1D nano-structure is a system with two dimensions are on the nano-scale. This results in a mono-dimensional system. It means that the system is developed along only one of the three spatial dimensions. In quantum physics, it might be though as a space region where quantum particles may travel only by following a one-dimensional track. For that reason, systems like that are called quantum-wires. Modern nanotechnology has provided us with several quantum-wires: silicon nano-wires [27] and carbon nanotubes [28] are good examples of systems where the majority of carriers are confined in a mono-dimensional space region. Carbon nanotubes are allotropes of carbon. They are highly organized carbon structures with cylindrical shapes. These carbon cylinders may be constituted by a single wall or have multi walls. In principle, carbon nanotubes might be seen as portions of graphene sheets rolled-up to obtain tubes, as schematically shown in Figure 5. Typically, their lengths are in the micro-scale while their diameters are in the nano-scale. Lengths are usually below 5 μm, while diameters are usually close to 2 nm for Single Walled and in the range between 2 and 15 nm for Multi-Walled Carbon Nanotubes. The latter may also reach lateral sizes of 60 nm or more. Consequently, the huge form-factor of carbon nanotubes makes them suitable to confine carriers in almost mono-dimensional shaped space regions. This confinement of majority carriers in a mono-dimensional space region, combined with the graphene-like conductance bands, results in amazing properties.

**Figure 6.**
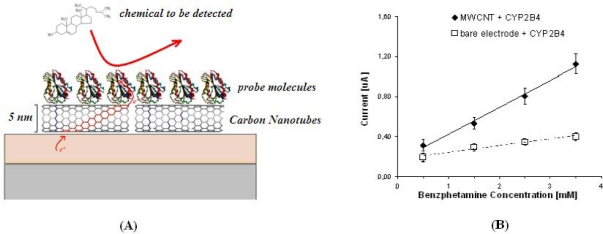
(A) Model describing the role played by carbon nanotubes in the electron-transfer between cytochromes and electrode. (B) Sensitivity enhancement due to carbon nanotubes in case of benzphetamine (a commonly used appetite suppressant drug) detected by the cytochrome P450 2B4 (reprinted from reference [36], with permission from IEEE Publishing, copyright 2009).

Carbon nanotubes present maximum current densities larger than 10^9^ A/cm^2^ [29], thermal conductivities over 3,000 W/mK [30,31], mean free-paths for charge carriers in the range of 1,000–25,000 nm [32,33]. They can be used as linear field emission sources [34] and their field-emission is enhanced by the adsorption of water molecules [35]. Thus, they are suitable candidates to promote electron-transfer processes from biochip electrodes to enzyme probes in water buffers. Figure 6(A) schematically shows the mediator action provided by carbon nanotubes in case of chemical detection using enzymes as probes. Enzymes are proteins which transform biochemical molecules (usually called enzyme’ substrates) by means of redox reactions. In such reactions, electrochemical species exchange electrons with electrode and it enables the detection. The enhancement of electron-transfer between probes and electrode results in an increased sensitivity.

Figure 6(B) shows this improvement in the case of benzphetamine detected by the enzyme P450 2B4 (also called CYP2B4). P450 belongs to a special category of enzymes, called cytochromes. The P450 family presents more than 3,000 different isoforms, which play a central role in all eukaryotic organisms, including humans. In fact, they are key-role proteins in any metabolic chain. Different isoforms have different substrates. In some cases, their substrates are endogenous compounds. In some other cases, the substrates are exogenous compound, e.g., pharmacological drugs. There are thousands of different P450 proteins, each of which may catalyze tens of different compounds. The cytochrome P450 2B4 also detects commonly-used anti-obesity drugs [36]. The isoform P450 2C9 may detect anti-inflammatory or anti-coagulant drugs [37]. The P450 3A4 may detect vasodilators or sedatives [38], or anti-cancer agents [36]. On the other hand, the P450 11A1 detects cholesterol [39], while the 4A11 detects testosterone [40], and the 2J2 detects arachidonic acid [41]. Thus, the above mentioned P450/compound couples show that there are plenty of opportunities to develop nano-bio-sensing applications by using enzymes from the P450 family. Moreover, the electron-transfer improvement due to carbon nanotubes has been already proven for the isoforms 11A1 [42] and 2B4 [36]. An enhanced sensitivity of 1.12 μA/mM mm^2^ was registered in cholesterol sensing by using multi-walled carbon nanotubes [42] while only 0.69 μA/mM mm^2^ was found by using electrodes with other molecular mediators [39]. Sensitivity in benzphetamine detection was enhanced up to 20.5 nA/mM mm^2^ by using multi-walled carbon nanotubes, while only 5.1 nA/mM mm^2^ was reached with bare electrodes [36].

Oxidases may be used to detect endogenous metabolites too. They are another kind of enzymes that catalyze redox reactions involving substrates related to human metabolism. Hydrogen peroxide is produced in such reactions. The peroxide releases two electrons to polarized electrodes. The electrons may be then counted for stoichiometric detection of the oxidase substrate. Glucose, lactate, glutamate, and other metabolic molecules may be detected choosing their proper oxidase. Electron-transfer enhancement up to two orders of magnitudes was demonstrated due to carbon nanotubes in case of hydrogen peroxide detection [43]. Similar gains in terms of sensitivity improvements have been obtained by using carbon nanotubes in biosensors based on oxidases. Sensitivity in glucose detection was enhanced up to 171,2 μA/mM cm^2^ [44] by using multi-walled carbon nanotubes, while only 15 μA/mM cm^2^ was obtained by using sol-gel films [45]. Sensitivity in lactate detection was improved to 2.1 [46], 8.3 [47], and 19.7 [43] μA/mM cm^2^ by using multi-walled carbon nanotubes, while only 0.24 μA/mM cm^2^ was reached by using titanate nanotubes [48].

These sensitivity enhancements are not only related to an increase of the effective area due to nano-structured morphology of the electrodes. Phenomena like the increase of capacitive currents [49], peaks enhancement in voltammetry [42], peak potential shifts [50], increased layering [51], and super-capacitance effects [52] take place, too.

## 4. 0D Nano-Structures Improving Enzymes-Chip

A 0D nano-structure is a system where all the dimensions are on the nano-scale. It might be seen as a 0-dimensional system, like a box with negligible size. In quantum physics, it might be though as a space region where quantum particles may be trapped.

**Figure 7.**
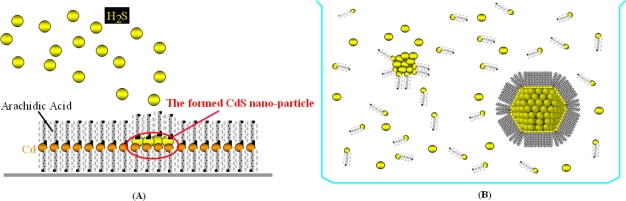
(A) Schematic model describing the role of hydrogen sulfide (H_2_S) in creating semi-conducting nano-particles inside a Langmuir-Blodgett mono-layer made of arachidic acid. (B) Schematic model describing the role of alkanethiols in stabilizing metallic nano-particles in a solution which contains gold salts (HAuCl_4_).

For that reason, such systems are called quantum-dots. Modern nanotechnology have provided us with quantum-dots by fabricating nano-particles with very simple chemical procedures. Although nano-particles may be fabricated by physical processes, they may be more easily grown by atom aggregation in liquid or quasi-liquid conditions and further stabilized by organic matrices. Here two different techniques will be briefly summarized: metallic-particle growth in liquid and semi-conducting particle growth in Langmuir-Blodgett films. In the latter case, an ordered mono-layer is obtained by compressing amphiphilic molecules at the air/water interface. Then, the layer is transferred onto a solid substrate by vertical dipping (Langmuir-Blodgett technique). Repetition of the transfer step results in multi-layers formed on a solid substrate. Multi-layers made by arachidic acid may be used to grow semi-conducting nano-particles within the film. An atmosphere of hydrogen sulfide is used to aggregate atoms into the arachidic acid matrix, as shown in Figure 7(A). By following this technique, semi-conducting nano-particles of CdS [53], PbS [54], and CuS [55] may be fabricated.

**Figure 8.**
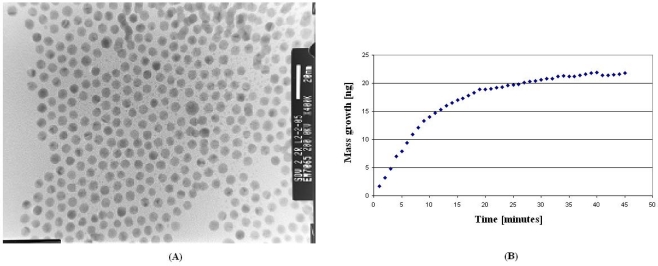
(A) SEM image of thiols capped gold nano-particles with size close to 5 nm. (B) Mass changing during nano-particles formation induced by injection of hydrogen sulfide (H_2_S) inside a Langmuir-Blodgett multi-layer made of cadmium arachidate.

Metallic nano-particles may be indeed formed under liquid conditions. Starting from metallic salts in solution, the particle formation proceeds till a nugget is formed when the solution reaches proper conditions for atom aggregation. To avoid big particles, alkanethiols are added into the salt solutions. As shown in Figure 7(B), thiols also aggregate forming cores. The alkyl chains create an organic shell surrounding the core, which provides a repelling coating to hinder further incoming atoms. The result is a solution of mono-dispersed metallic nano-particles. Is has been shown that gold [56], silver [57], rhodium [58], platinum and ruthenium [59] nano-particles may be fabricated by using this simple technique. Most importantly, the size of such particles may be precisely driven by changing the salts-to-thiols ratio. Gold nano-particles were precisely fabricated with different diameters from 1.5 to 5.2 nm by adjusting such ratio [60], as demonstrated by Scanning Electron Microscopy results shown in Figure 8(A).

This technique is also known with the name of “Brust method”, after the first scientist who proposed this procedure to obtain colloidal mono-dispersed gold nano-particles under liquid conditions [61]. The two above-mentioned techniques are suitable for fast, easy, low-cost fabrications of 0D nano-structures with conducting or semi-conducting character. They are low-cost since they require only simply organic chemistry equipment. Large and expensive under-vacuum systems are avoided in these fabrication processes. They are easy because only simple and safe chemical protocols are envisaged. They are fast because the required production time is on the hours or minutes scale. Figure 8(B) shows mass growth during nano-particles formation within forty multi-layers of cadmium arachidate. All the CdS particles are formed in the organic matrix within 35 minutes. Semi-conducting particles mono-dispersed in solution may be obtained by removing the arachidic matrix with commonly used solvents.

**Figure 9.**
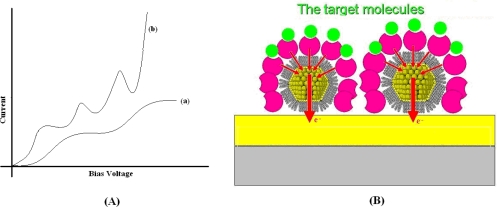
(A) Schematic curves showing staircase curve (a) and curve with oscillations (b) related to charge trapping within a quantum-dot. (B) Schematic model describing the role played by thiols capped gold nano-particles in electron-transfer between cytochromes and electrode.

These extremely small particles are good quantum-dots able to trap conducting carriers. The theory of Coulomb Blockade, developed by Averim and Likharev in the ’80s [62], foresees that electrons may be trapped in a quantum-dot if the electrostatic energy keeping them in the dot is larger than its thermal excitation. If the electrostatic energy is smaller, then the electrons might drop out from the dot. A simple semi-classical explanation may give an idea of the system physics. When an electron has enough energy to be trapped inside the dot, the further incoming electron is under its electrostatic repulsion. Thus, we might expect a current suppression in a current/voltage curve for those bias voltages close to each single trapping event. Therefore, current/voltage curves look like curve (a) in Figure 9(A), which presents steps equally distributed in voltage. Such curves were initially registered at very low temperature [63] and successively at room temperature in smaller particles [64]. This is due to the relationship between the electrostatic energy and the dot size. The smaller the dot size is, the larger the electrostatic energy of trapped electrons is, and easier it is to overcome the thermal excitation, even at room temperature. Equally distributed oscillations were also observed at room temperature [65], similar to those reported in curve (b) of Figure 9(A). They were related to Coulomb Blockade in nano-clusters, too. The shift between curve (a) and curve (b) on the same nano-particle has been demonstrated by varying the tunneling barrier between the probe tip and the particle [66] or by varying the particles size [67].

Such kinds of carrier trapping may be used to enhance charge storage, current transport, and electron-transfer in systems structured with quantum-dots. For example, Figure 9(B) schematically shows how gold nano-particles may be used as mediators to enhance the electron-transfer between cytochromes and electrodes. A gain in terms of sensitivity of 6.5 μA/mM mm^2^ in cholesterol detection by using cytochromes P450 and gold nano-particles of 12 nm has been already demonstrated [68]. Similar improvements in sensitivity were also obtained in oxidase-based biosensors: 16.5 μA/mM mm^2^ for glucose [69], 500 μA/mM mm^2^ for lactate [70], and 70.4 μA/mM mm^2^ for phenol [71] by working with electrodes structured with nano-particles.

## 5. New Electrochemical Systems for Personalized Therapies and Cell Biology

In personalized therapy, one key-strategy is to supply drugs to patient in the right amount by monitoring disease progress, drug efficacy, and the patient’s metabolism. For this reason, precise measurements of bio-markers and drugs concentration in patient’s blood are highly required.

The squamous cell carcinoma antigen (SCCA) bio-marker is over-expressed in hepatocarcinoma with concentrations in serum from 14.9 fM to 18.9 fM [72]. The α-fetoprotein is also over-expressed in hepato-cellular carcinoma with serum concentrations close to 0.7 nM [73]. The possibility to detect close to fM concentration ranges has been already demonstrated by using a capacitance-based technique in sensing of metal ions [74]. A fully-electronic capacitance based bio-chip was also developed [10]. However, low specificity [10,19], low reproducibility [10,17–19], very large time-drift [17,20] all seriously affect the applicability of this technique. New solutions from nano-bio-science were recently demonstrated by using probe surfaces structured with 2D glycol nano-layers and they could pave the way for improved portable device systems in cancer markers detection [25].

Therapeutic values of drug concentration in blood or plasma are different on a drug-by-drug basis. Typically they go from a few nM up to hundreds of μM. Cyclophosphamide, an anti-cancer agent, varies from 2.6 μM up to 76.6 μM [75]. Erythromicin, a well known macrolide antibiotic, ranges from 13.6 nM up to 68 μM [76]. Triazolam, a quite famous sedative to treat insomnia, varies from 0 to 10 nM [77]. Cyclosporin, one of the possible anti-inflammatory drugs, goes from 160 nM to 490 nM [78]. Moreover, patient-to-patient responses to nortriptyline vary from 2 μg/L up to 15 μg/L for the same injected amount of drug [1]. Thus, precise measurements of large concentration ranges are strictly required to succeed in developing bio-chips for drug monitoring in personalized therapy. Electrochemical detection of drugs was already investigated by using P450 cytochromes. It is also suitable for on-line monitoring, as enzymes are suitable for continuous catalysis of chemical reaction. However, the concentration range of a sensor for detection of verapamil, an antihypertensive drug, was from 400 μM to 3 mM [38] while its therapeutic range is below 0.3 μM [79]. Thus, new solutions from nano-bio-science are required to improve bio-chip sensitivity in order to reach the required drug concentration ranges and to develop fully-electronic bio-chips to be fruitfully used in personalized therapy [36].

Key-metabolites of cellular metabolism are glucose and lactate. The first is the “cellular fuel”, while the second is often associated to cell suffering. More important, they are sometimes cross-correlated and their detection can be important to understand cellular phenomena. For example, it has been assumed that neurons may use lactate produced by glia in case of glucose deprivation [80]. Such a model is actually important to understand what happens, e.g., in the case of brain ischemia. Moreover, other metabolites such as ethanol and galactose were recognized as important biomarkers of cellular metabolism [81]. Detection technologies available in biology laboratories to sense glucose and lactate are usually based on spectrophotometry with working range in hundreds of μM while glucose concentration in cell cultures may vary from 20 mM down to few mM during cell proliferation. The instrumentation is bulky and costly, and the tests are time-consuming. Moreover, spectrophotometer assays are not suitable for real-time monitoring, since they require off-line measurement of samples because of the chemical processing with colorimetric substrates. On the other hand, many different oxidases and dehydrogenases are naturally available and they may be used to develop low cost, highly integrated, continuous monitoring sensors. For example, glucose oxidase is widely used in both portable and implantable biosensors for diabetic patients. Therefore, improved solutions to develop fully-electronic bio-chips for real-time monitoring in cell cultures are now possible by integrating enzymes for continuous monitoring, 1D or 0D nano-structures for sensitivity enhancement, and novel CMOS designs to improve chip functionalities [50].

## 6. Conclusions

In this paper, we have seen different available technologies for building nano-structures where one, or two, or all the three physical dimensions are at the nano-scale. By following the well-know conventional terminology, we can refer to those systems as 2D, 1D, and 0D systems because they are developed only in 2, or, 1, or 0 dimensions. We have seen different examples of such nano-systems, like highly-ordered and densely-packed organic mono- and multi-layers (2D structures), single-walled and multi-walled carbon nanotubes (1D), semiconducting and conducting nano-particles embedded in organic matrices (0D). All the above mentioned nano-structures provide new functionalities with respect to the same material structured at the macro-scale. Highly-packed ethylene-glycol 2D nano-layers have shown amazing stable electrical properties suitable for highly reproducible capacitance bio-detection with improved specificity. 1D nano-tubes made of carbon atoms have shown amazing electrical conductivities resulting in sensitivity enhancements when applied to bio-detection. 0D nano-particles made of metallic or semi-metallic materials have been used to trap electrical conductive carriers, which results in an improved sensitivity when applied to bio-detection. The comparison between the emerging demands in biomedicine (e.g., detection ranges) and the performances of state-of-the-art bio-sensors (e.g., detection limits) clearly shows that nanotechnology may widely contribute in developing new electrochemical systems for monitoring in personalized therapy and cell biology.
